# Supplementary material for: Methods for quantitative susceptibility and R2* mapping in whole post-mortem brains at 7T applied to amyotrophic lateral sclerosis
Source: Neuroimage. 2020 Nov 15;222:117216. doi: 10.1016/j.neuroimage.2020.117216 (PMC7775972; doi:10.1016/j.neuroimage.2020.117216)
Supplement: Supplementary file 1 [file mmc1.pdf]

Supplementary material

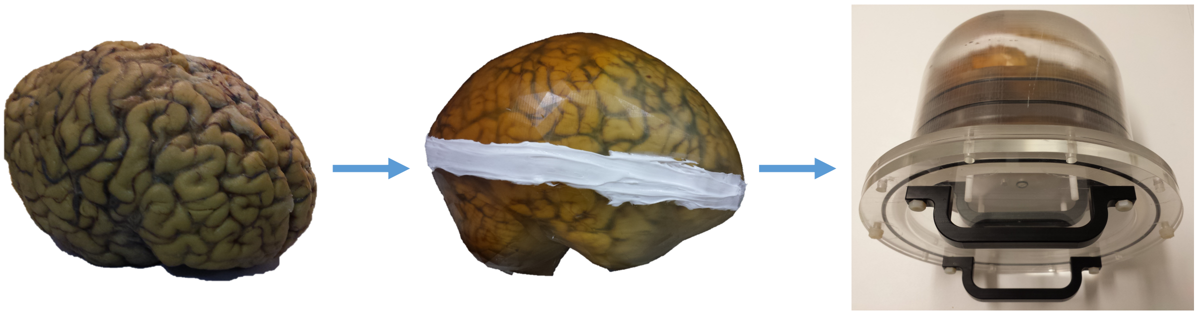

Fixed post-mortem brain **3D printed shell** **Custom holder**

Figure S1 Illustration of the 3D printed shell and custom holder for scan preparation. The 3D printed shell was provided by Dr Alard Roebroeck, Maastricht University.

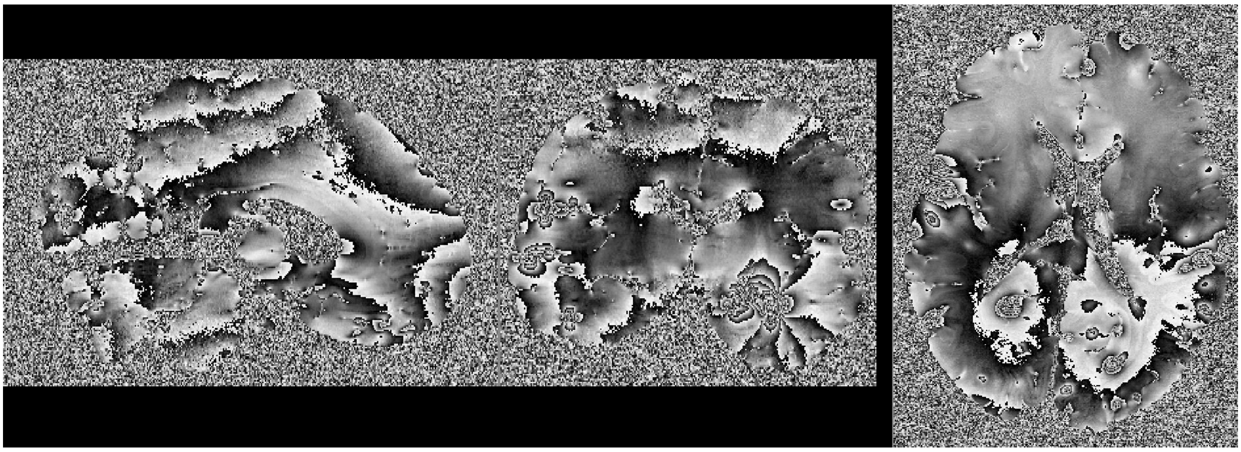

3rd echo phase image from GRE acquisition
with spherical holder

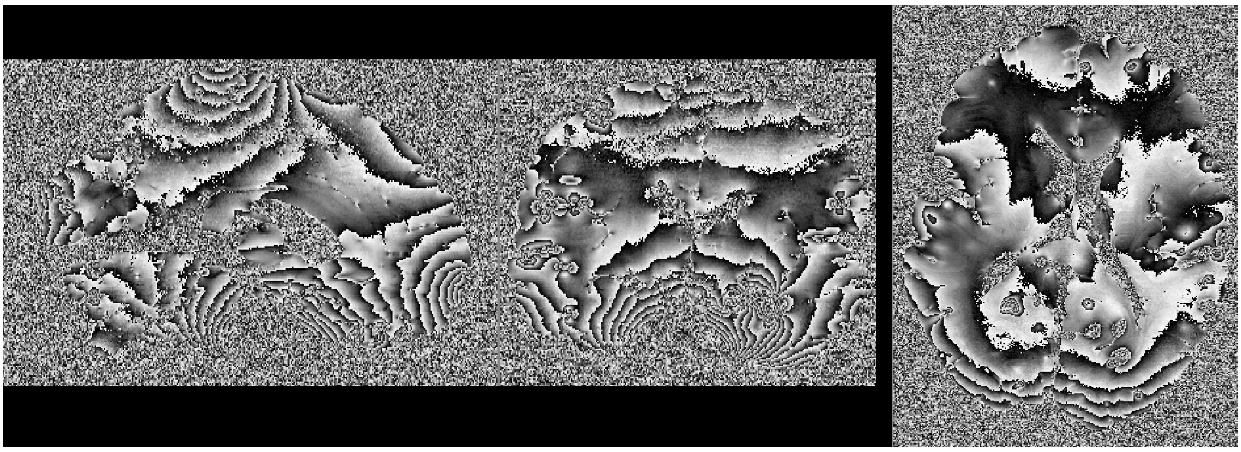

3rd echo phase image from GRE acquisition
without spherical holder

Figure S2 Comparison of the measured GRE phase in a post-mortem brain with (top) and without (bottom) the custom holder (Supplementary material Figure S1). The phase images acquired with the spherical holder (top) show substantially less phase aliasing, reflecting improved B_0 homogeneity. Phase images displayed here generated using the manufacturer-supplied reconstruction, rather than the custom reconstruction pipeline from the k-space data described in the main text.

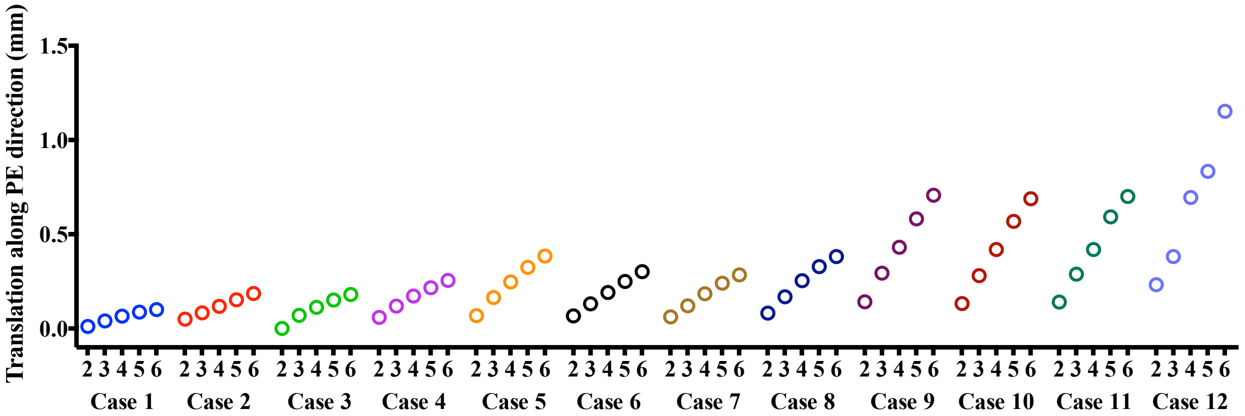

Figure S3 Translations along the phase encoding direction estimated to register the 1st echo to the 2nd - 6th echoes for repeat 1 using FLIRT for all cases. The slice thickness was 1.2 mm for Case 1-7 and 0.6 mm for Case 8-12, leading to longer scan time per GRE repeat for Case 8-12 and hence larger misalignments along the phase encoding direction. Note translations shown here were calculated using the scanner reconstructed magnitude data, hence values might be slightly different to the actual translations applied in our custom processing pipeline described in the main text. All four repeats were available for case 2 using this reconstruction method.

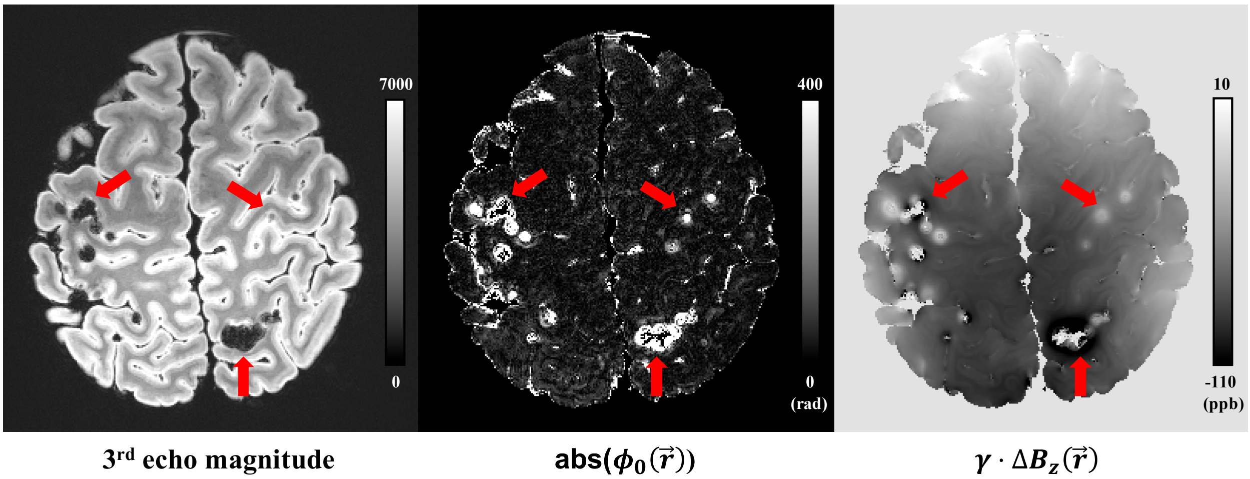

Figure S4 Illustration of detecting noisy voxels (with unreliable phase that correspond to air bubbles or blood vessels) from the $\phi_0(\vec{r})$ map estimated from fitting. Air bubbles and blood vessels correspond to large, focal voxels on the $abs[\phi_0(\vec{r})]$ maps (red arrows).

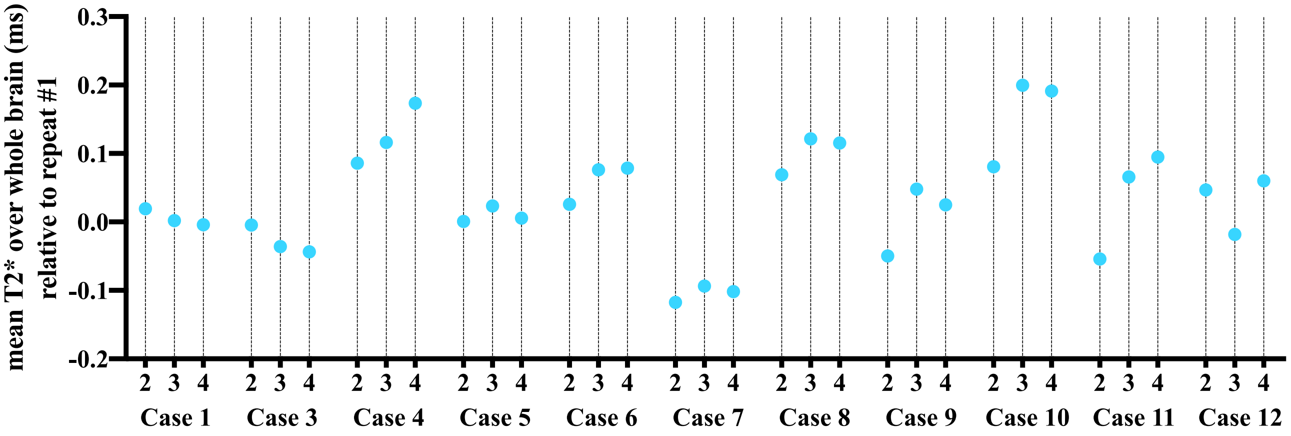

Figure S5 Change in T2* over the whole brain for repeats 2-4 relative to repeat 1 in 11 brains. Only two repeats were available for case 2, which was not included here.

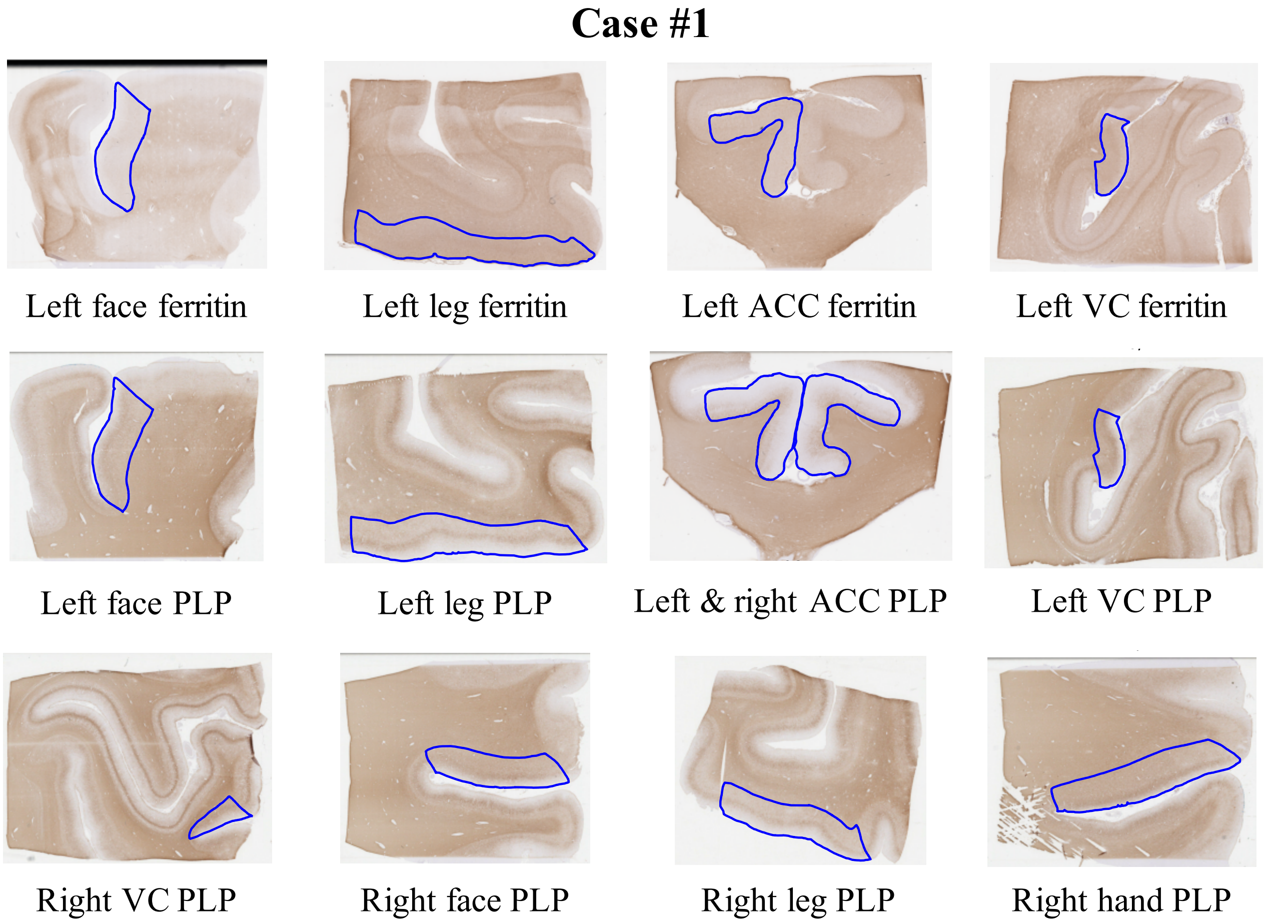

Figure S6 Digital stained histology images with cortical annotations for Case 1.

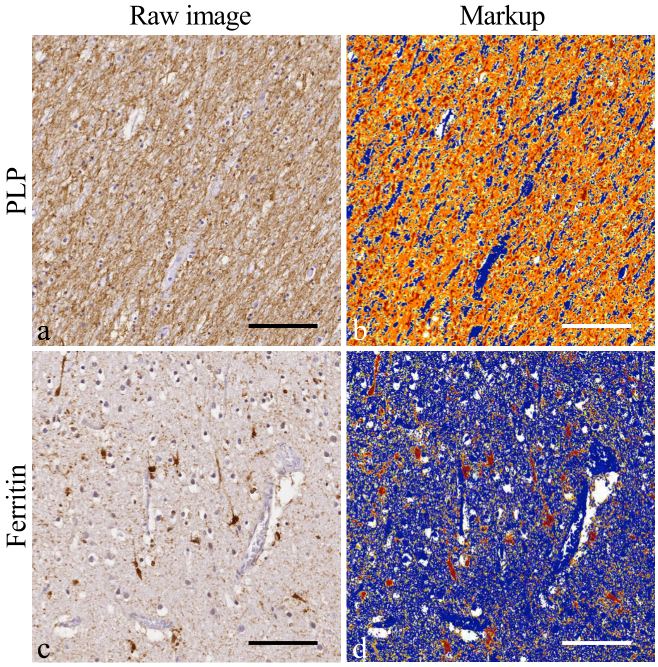

Figure S7 Digital histology images from motor cortex subcortical white matter stained for PLP (a) and grey matter stained for ferritin (c) in ALS. Colour deconvolution for positive staining (brown) generates markup images (b and d) showing unstained pixels as white, negative stained pixels as blue, weak positive pixels as yellow, medium positive pixels as orange and strong positive pixels as red.

Scale bar = 100 µm

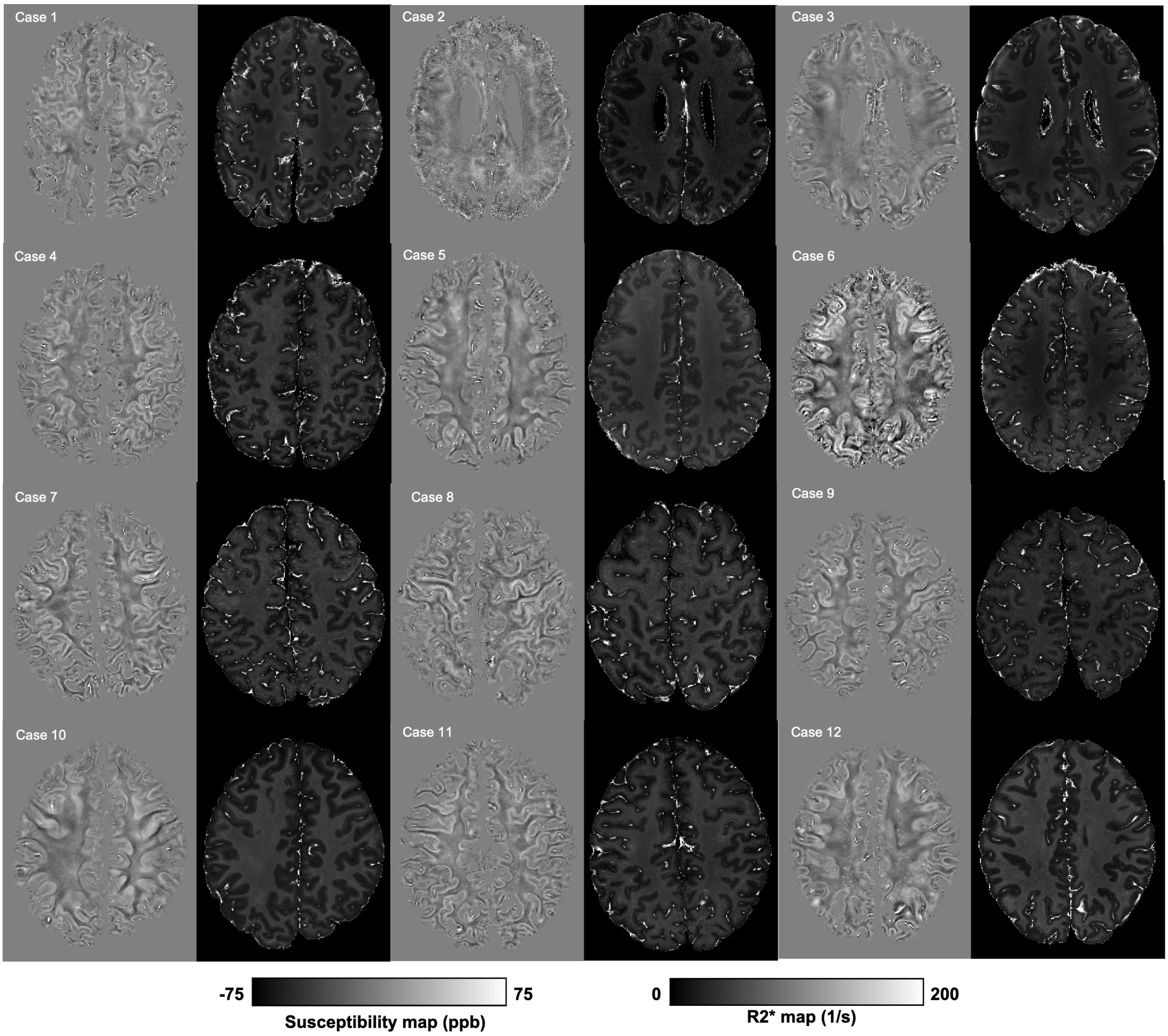

Figure S8 R2* and quantitative susceptibility maps from all brains.

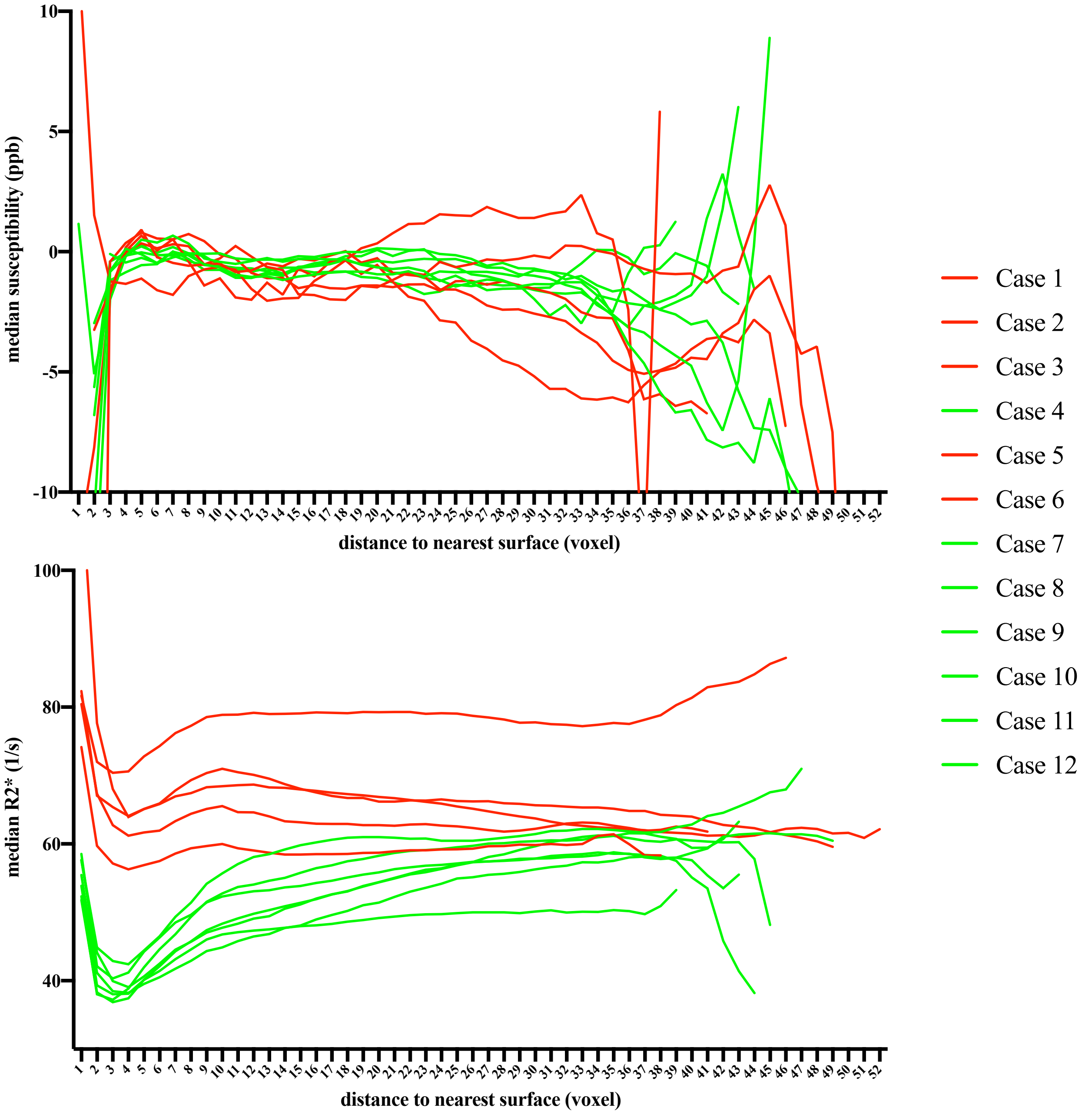

Figure S9 Median susceptibility and R2* of voxels with different distances to the nearest brain surface for all thirteen brains. Here the red and green lines correspond to the two different fixatives used within our project (red - 10% formalin and green - 10% neutral buffered formalin). For the R2* maps, a difference is observed for the R2* estimates which depends on the type of fixative used. A similar trend is not observed for the susceptibility estimates.
